# Substitution Patterns Can Limit the Effects of Sugar-Sweetened Beverage Taxes on Obesity

**DOI:** 10.5888/pcd10.120195

**Published:** 2013-02-07

**Authors:** Jason Fletcher, David Frisvold, Nathan Tefft

**Affiliations:** Author Affiliations: David Frisvold, Emory University, Atlanta, Georgia; Nathan Tefft, University of Washington, Seattle, Washington.

Dramatic increases in obesity and sugar-sweetened beverage consumption over the past several decades have become major public health and clinical concerns. Obesity rates tripled in 30 years, and sugar-sweetened beverage consumption among children more than doubled in the last 2 decades of the twentieth century (1). Many children drink more sugar-sweetened beverages than milk, and sugar-sweetened beverages represent the largest category of daily caloric intake (7%–12%) for many demographic groups (1). Emerging evidence suggests that increasing consumption of sugar-sweetened beverages raises weight and obesity rates.

These trends and the evidence that modest but persistent reductions in calories (approximately 1 can of sugar-sweetened beverage per day) could halt the obesity epidemic for 90% of the population or more (2) has focused attention toward enacting policies to curb consumption of sugar-sweetened beverages, especially in children. Most prominent among these policies has been the push to increase the prices of sugar-sweetened beverages through increases in state and local taxes on these items. A recent Institute of Medicine (IOM) report suggests that governments implement a sizeable tax to reduce the overconsumption of sugar-sweetened beverages (3). Originally proposed more than a decade ago (4), the idea follows basic economic reasoning and the compelling success story of the reduction in tobacco use from tobacco taxation. The economic intuition is that higher prices discourage use, so enacting a “sin tax” on carbonated soft drinks (or sugar-sweetened beverages in general) could lower consumption; subsequently, the effect of decreased sugar-sweetened beverage consumption on obesity rates could also have an effect on the high social costs of obesity.

Although U.S. states have taxed carbonated soft drinks for nearly 100 years as a means of raising revenue, only recently has this policy been evaluated for its potential effect on reducing obesity rates. Evidence on this issue is available primarily in 2 varieties. A host of studies have used data on household consumption and grocery store prices to show that households that encounter higher prices reduce carbonated soft drink purchases (5). These estimated price responses are often used by other researchers to calculate the reductions in calories attributable to lower carbonated soft drink consumption and to then calculate the implied reduction in weight from these reductions in calories (6). A typical estimate is that a 10% price increase on carbonated soft drinks would reduce consumption by approximately 10%, leading to a 20 kcal per day reduction (7).

These studies do not fully address several complex behavioral and biological responses, however. These responses include the possibility that consumers would substitute other caloric beverages (eg, orange juice, chocolate milk) or foods for sugar-sweetened beverages if only the latter are taxed. Another possibility is that manufacturers would respond to higher taxes by lowering prices (“strategic pricing”). A third possibility is a dynamic response of weight to a change in the rate of sugar-sweetened beverage consumption. A second variety of studies addresses these issues by directly linking existing state-level carbonated soft drink tax rates to information about both daily beverage consumption and measured weight to estimate actual (as opposed to hypothetical) tax effects. These studies have found little or no evidence that people faced with higher carbonated soft drink taxes have lower weight (8).

How could these 2 sets of studies come to such different conclusions on the effects of carbonated soft drink taxes on weight? A key distinction between these similar approaches is in how substitution patterns are incorporated — that is, if consumers drink fewer carbonated soft drinks, what, if anything, do they drink instead? Studies of the first variety typically assume that consumers respond only partially to the reduction in carbonated soft drink calories by increasing intake of other caloric beverages such as juice or whole milk. For example, some researchers (6) assume only one-third of each calorie reduction in carbonated soft drinks from taxation is replaced by substitution of other high calorie beverages. Rather than make these assumptions, studies of the second variety estimate actual substitution patterns and have shown that consumers fully offset all calorie reductions in carbonated soft drinks from taxation by drinking other high-calorie beverages. These studies find no reduction in population weight from carbonated soft drink taxes.

On the basis of substantial increases in both obesity and sugar-sweetened beverage consumption in the past decades and the links between them, the effectiveness of sugar-sweetened beverage taxation in reducing rates of obesity is essential to consider in any discussion of public health measures available to reduce obesity rates. Evidence suggests caution in enacting sugar-sweetened beverage taxation legislation with a core purpose of obesity reduction. “Big taxes” may have different effects than the small taxes currently in place, but these big taxes would need to dramatically shift the substitution patterns we see when small taxes are implemented. Big taxes would need to effectively shift people toward water and other low-calorie drinks in a way that small taxes do not; taxes alone would not likely cause such a shift.

However, what if sugar-sweetened beverage taxes are ineffective in reducing obesity? Does this mean that we should not consider implementing them as public health measures? Not necessarily — the broader benefits of tax increases on sugar-sweetened beverages must be considered. Most studies suggest that such taxes will likely reduce consumption of sugar-sweetened beverages, and researchers (8) find that the reduction in calories from soft drinks is equivalent to the increase in calories from whole milk, which may be a good public health outcome, irrespective of any obesity reduction. Associated high levels of added sugar may also lead to other poor health outcomes, such as diabetes, cardiovascular risk, and poor dental health. Sugar-sweetened beverage taxes could successfully combat these poor health outcomes even if taxation does not lower obesity rates.

In addition to encouraging other potential health benefits, sugar-sweetened beverage taxes may be helpful in reducing obesity rates if they are used as one aspect of a larger, more comprehensive policy approach that aims to redirect consumers away from caloric sweeteners and toward more healthful alternatives such as water or food without added sweeteners. For example, a tax combined with a subsidy for water could be more effective than a tax in isolation. Additionally, a recently proposed extension of a sugar-sweetened beverage tax that instead taxes all caloric sweeteners at the manufacturer level would limit consumers’ ability to easily switch to foods with caloric sweeteners or other unhealthful beverages (9). A key feature of this broader approach is that it limits the possibility for substitution to unhealthful alternatives, ensuring that these policies effectively achieve the goal of reducing and stemming the rise in obesity.
